# Multivariate associations between systemic inflammation, metabolic dysregulation, and cognitive performance in project FRONTIER

**DOI:** 10.3389/fnagi.2026.1836968

**Published:** 2026-07-14

**Authors:** Chathurika S. Dhanasekara, Chanaka N. Kahathuduwa, Volker Neugebauer

**Affiliations:** 1 Department of Pharmacology and Neuroscience, School of Medicine, Texas Tech University Health Sciences Center, Lubbock, TX, United States; 2 Garrison Institute of Aging, School of Medicine, Texas Tech University Health Sciences Center, Lubbock, TX, United States; 3 Center for Translational Neuroscience and Therapeutics, School of Medicine, Texas Tech University Health Sciences Center, Lubbock, TX, United States; 4 Department of Neurology, School of Medicine, Texas Tech University Health Sciences Center, Lubbock, TX, United States

**Keywords:** biomarkers, cognitive functioning, executive functions, inflammation, metabolic marker

## Abstract

**Background:**

Cognitive aging is heterogeneous, and growing evidence implicates chronic low-grade inflammation and metabolic dysregulation in neuropathology and cognitive decline. This study examined the multivariate relationship between inflammatory-metabolic biomarkers and neuropsychological performance in a large, community-based cohort of adults from rural West Texas enrolled in Project FRONTIER.

**Methods:**

Participants aged ≥ 40 years who completed a study visit in the Project FRONTIER study were included (*n* = 1,357). Associations between systemic metabolic health and cognitive performance were examined using a domain-driven metabolic marker set and a cognitive variables set. Canonical correlation analysis (CCA) was used to assess shared variance between biomarker and cognitive domains, followed by structural equation modeling (SEM) using variables with the highest canonical loadings as an exploratory model. Analyses were conducted in R version 4.5.2.

**Results:**

CCA revealed a significant multivariate association between inflammatory-metabolic biomarkers and cognitive performance. The first canonical correlation was 0.242 (95% CI: 0.171–0.310), accounting for 5.8% of the shared variance between the biological and cognitive domains. This association was statistically significant based on both Wilks’ lambda (*p* = 1.05 × 10^–6^) and permutation testing (Fisher combined *p* < 0.001) and remained stable in bootstrap validation (*r* = 0.255, 95% CI: 0.219–0.294, 5,000 samples). Among biomarkers, C-reactive protein showed the strongest loading (0.69), followed by gamma-glutamyl transferase (0.28), fasting blood sugar (0.21), abdominal circumference (0.20), and hemoglobin A1c (-0.18). Among cognitive measures, EXIT-25 (loading = −0.59) showed the strongest contribution, followed by Trail Making Test Part B (loading = −0.45), RBANS-visuospatial/constructional (loading = −0.41), clock drawing (loading = −0.31), and RBANS-Language (loading = −0.30). SEM showed acceptable fit and further supported the finding that a higher inflammatory-metabolic burden was associated with worse executive function after adjusting for age, sex, ethnicity, education, and income (β = −0.310, standardized β = −0.202, *p* < 0.001).

**Conclusion:**

Systemic inflammation and metabolic dysregulation were associated with poorer cognitive performance, particularly executive dysfunction, in this rural aging cohort. These findings, while modest in effect size, support the importance of cardiometabolic health in cognitive aging, motivating future longitudinal and mechanistic studies.

## Introduction

While cognitive aging is a heterogeneous process, a growing body of evidence implicates systemic physiological processes, particularly chronic low-grade inflammation and metabolic dysregulation, as key drivers of neuropathology and cognitive decline (Sartori et al., 2012; van den Kommer et al., 2012). However, the evidence currently available on these factors, especially inflammation, is heterogeneous and equivocal (Dyer et al., 2024; van den Kommer et al., 2012). Despite the compelling evidence linking individual biological markers to cognitive health, a significant limitation of prior research is the tendency to examine these factors in isolation (Chrzanowski et al., 2025; Lin et al., 2023; Long et al., 2023; Ravaglia et al., 2007; van den Kommer et al., 2012; Wersching et al., 2010). Inflammation and metabolic dysregulation are not independent processes; they are deeply intertwined, often co-occurring and synergistically contributing to end-organ damage. Consequently, analytical approaches that consider only single biomarkers fail to capture the complex, multidimensional nature of these systemic risks. To address this gap, multivariate statistical methods are needed to model the collective influence of a broad array of biological markers on the multifaceted construct of cognition.

Canonical Correlation Analysis (CCA) is a powerful multivariate statistical technique well-suited for this purpose. CCA identifies the optimal linear combinations of variables from two distinct sets (Hotelling, 1992). This data-driven approach allows for the characterization of the shared variance between biological and cognitive domains, revealing underlying patterns of association that might be obscured in univariate analyses. Its application in neuroscience has grown, proving effective in exploring complex brain-behavior relationships and identifying covariance patterns in aging and disease (Dyer et al., 2024).

Project FRONTIER, a large community-based cohort from rural West Texas, provides a rare opportunity to examine these multivariate associations in the population where cardiometabolic risk may matter most. Studying these associations in a rural, underserved population is critical, as such communities often face unique health challenges and may exhibit different risk profiles. For instance, rural, medically underserved populations bear a disproportionate burden of cardiometabolic disease and limited healthcare access, yet are markedly under-represented in cognitive-aging cohorts (Appiah et al., 2022; Miller et al., 2023). Therefore, the present study leverages CCA to investigate the multivariate relationships between a comprehensive panel of inflammatory and metabolic biomarkers and a detailed battery of neuropsychological assessments in a cohort of middle-aged and older adults. We hypothesized that a significant, positive canonical correlation would emerge, revealing a primary dimension of shared variance linking a profile of elevated inflammatory and metabolic burden to a pattern of diminished cognitive performance, with a particular impact on executive function.

## Materials and methods

### Participants

This study was conducted using data from an ongoing longitudinal study of aging adults in rural West Texas, Project FRONTIER (Facing Rural Obstacles to Healthcare Now Through Intervention, Education and Research) (O’Bryant et al., 2011). Individuals enrolled in Project FRONTIER were ≥ 40 years of age and residents of either Cochran, Bailey, Hockley, or Parmer County, Texas. All participants signed written informed consent prior to participation in the study, and the study protocol was approved by the Institutional Review Board at Texas Tech University Health Sciences Center (Legacy-L06-028-U). Participants underwent a medical examination in their county of residence, a neuropsychological test battery, and an in-depth interview. A detailed description of the cohort and community-based participatory research procedures has been published elsewhere (O’Bryant et al., 2009; O’Bryant et al., 2011). For the present analysis, we included participants who had completed their first study visit. Thus, the final sample comprised 1,357 participants.

#### Variable sets

The cognitive variable set included the Repeatable Battery for the Assessment of Neuropsychological Status (RBANS) (Randolph et al., 1998) and its five indices (domain) scores (Immediate Memory index: RBANS-IM, Delayed Memory index: RBANS-DM, attention: RBANS-Attention, language: RBANS-Language, visuospatial/constructional abilities: RBANS-Visuospatial), Executive Interview 25 (EXIT-25) (Royall et al., 1992), verbal fluency (FAS) (Borkowski et al., 1967), Clock Drawing Test (CLOX) (Royall et al., 1998), Trail Making Test Part A and B (TMT-A and TMT-B) (Army Individual Test Battery, 1944; Ashendorf et al., 2008; Reitan, 1958). These assessments have shown validity and reliability in measuring executive functioning, attention, language, immediate and delayed recall, and visuospatial/constructional ability.

A domain-driven set of metabolic markers was selected to examine associations between systemic metabolic health and cognitive performance. This metabolic marker set comprised indices of chronic and short-term glycemic control (hemoglobin A1c-HbA1c, fasting blood glucose-FBS), (Lin et al., 2023; Saczynski et al., 2008) lipid metabolism (triglycerides, high density lipoprotein-HDL), (Solomon et al., 2009a; Solomon et al., 2009b) nutritional status and adiposity [albumin, (Cui et al., 2024) body mass index-BMI, (Elias et al., 2005; Li et al., 2020), abdominal circumference (Norris et al., 2023)], hemodynamic load [systolic blood pressure-SBP (Kałamała et al., 2025; Williamson et al., 2019), systemic low-grade inflammation [C-reactive protein-CRP (Long et al., 2023; Ravaglia et al., 2007; van den Kommer et al., 2012), an acute-phase reactant synthesized by the liver], renal function [creatinine (Gasparini et al., 2026)], and hepatic/metabolic stress and oxidative stress [gamma-glutamyl transferase, GGT, a hepatobiliary enzyme, circulating GGT is increasingly recognized as an early indicator of oxidative stress and cardiometabolic risk (Chen J. et al., 2024; Zhong and Li, 2025)]. These markers were selected because each has been robustly linked to cognitive impairment, dementia risk, or relevant cerebrovascular/structural brain changes in epidemiologic and clinical cohorts, and together they capture complementary dimensions of cardiometabolic risk, inflammation, and end-organ damage that plausibly influence late-life cognition via vascular, neurodegenerative, and oxidative stress mechanisms (Chen M. D. et al., 2024; Han et al., 2025; Huang et al., 2024; Zhong and Li, 2025).

### Statistical analysis

Prior to imputation, distributional properties of all continuous variables were examined using skewness statistics. Variables exhibiting substantial right-skewness (skewness > 1.0) were log-transformed to improve normality and linearity of relationships. Fasting glucose was winsorized at the 1st and 99th percentiles to address extreme outliers while preserving the bulk of the distribution. Multiple imputation was used to address missing data under the missing-at-random (MAR) assumption. Twenty imputed datasets were generated using the “mice” package in R (Van Buuren and Groothuis-Oudshoorn, 2011). All cognitive and biological variables were residualized with respect to demographic covariates (age, sex, education, income, and ethnicity, coded as Hispanic vs. non-Hispanic) using linear regression. Race was not modeled separately because the cohort was racially homogeneous (≈ 94% White).

### Canonical correlation analysis (CCA)

CCA was performed separately on each imputed dataset. Canonical correlations were pooled using Fisher’s z-transformation with Rubin’s rules to obtain combined estimates and 95% confidence intervals. Structure loadings (correlations between original variables and canonical variates) were averaged across imputations. To address potential sign indeterminacy of canonical variates across imputations, loadings were aligned to ensure consistent directionality before averaging. Sign stability was calculated as the proportion of imputations in which each loading of each variable matched the sign of the pooled loading. Statistical significance was assessed using two complementary approaches. First, Wilks’ Lambda (Λ) with asymptotic F-approximation was computed for each imputed dataset, and median *p*-values were reported. Second, permutation testing (5,000 permutations per imputation) was conducted, with *p*-values combined across imputations using Fisher’s method. To evaluate the internal reproducibility of the leading CCA dimension, we performed repeated five-fold cross-validation of the canonical correlation for completed data sets.

Redundancy analysis was performed to quantify the proportion of variance in each variable set explained by the canonical variates of the opposite set (Karpman, 1981). Statistical significance of the redundancy indices was evaluated using permutation testing (1,000 permutations per imputation). Sensitivity analyses were conducted to assess the robustness of the primary CCA. The CCA was repeated after excluding participants with dementia or mild cognitive impairment, with additional adjustment for county of residence, and separately within lower- and higher-income subgroups.

#### Structural equation model

We selected indicators based on the largest absolute structure coefficients on the primary canonical dimension (the first canonical function), prioritizing variables that were consistently strong contributors across imputations. The CCA-informed indicators were then carried forward to the SEM as an exploratory model. Variables with the strongest canonical loadings were retained to define two latent constructs: InflamMetab, an inflammatory-metabolic burden latent factor (CRP, GGT, FBS, and abdominal circumference), and ExecFunc, an executive dysfunction latent factor (EXIT-25, TMT-B, RBANS-Visuospatial, and CLOX). Cognitive indicators were retained on their native scales; however, for the TMT A and B (longer completion times indicate worse performance) and the EXIT-25 (higher scores indicate greater executive dysfunction), higher raw scores indicate poorer performance. To express higher values indicating better performance across all cognitive measures, these three measures were reverse-coded. Two residual covariances were specified a priori on substantive grounds and retained between RBANS-Visuospatial and CLOX, reflecting shared visuoconstructional/graphomotor variance, and between FBS and abdominal circumference, reflecting shared adiposity-glycemic metabolic variance. We adjusted for potential confounding by regressing both latent variables on age, sex, ethnicity, education, and income. We included direct paths from age to TMT-B (reverse-scored) and EXIT-25 (reverse-scored) in the primary SEM to account for indicator-specific age effects (i.e., differential item functioning) that are not fully captured by the latent executive function construct (Cheng et al., 2016). Both measures are particularly sensitive to age-related influences such as processing speed, motor/visual scanning demands, and task-specific administration effects; allowing these direct effects to be controlled helps ensure that the latent executive factor reflects the shared executive construct rather than residual age-related method variance.

The SEM was fit across imputed datasets using robust maximum likelihood with sandwich standard errors, and pooled inference followed Rubin’s rules. Models were estimated using robust maximum likelihood (MLR). Model fit was evaluated using the comparative fit index (CFI ≥ 0.90), Tucker–Lewis index (TLI ≥ 0.90), root mean square error of approximation (RMSEA ≤ 0.05) with 90% confidence intervals, and standardized root mean square residual (SRMR < 0.08) (Hu and Bentler, 1999). Modification indices were inspected to identify localized areas of misfit, with any added residual covariances required to have clear substantive justification (e.g., shared method variance among similar cognitive measures). Standardized estimates were reported alongside unstandardized estimates. Parameter estimates were obtained in each imputed dataset and pooled across imputations. All analyses were conducted in R using lavaan (v0.6-21) and lavaan.mi. All analyses were conducted in R version 4.5.2 using the mice, CCA, and CCP packages. Statistical significance was set at α = 0.05. Detailed methodology is included in Supplementary section.

## Results

### Characteristics of whole sample

Of 1,357 participants with a first study visit, all were retained for analysis under multiple imputation. Mean age was 59.2 years ± 12.2 (range 40–97; 67.9% aged 40–64 years and 32.0% aged ≥ 65 years). There were 68.2% (*n* = 922) females, and 59.9**%** (*n* = 805) were Hispanic. Concerning cognitive status, dementia: *n* = 26 (1.9%); MCI: *n* = 214 (15.8%). Complete data on all analytic variables were available for 645 participants (47.5%). Per-variable missingness ranged from 0.1% (age) to 41.4% (C-reactive protein); all variables other than CRP and Trail Making Test B (13.1%) were missing in fewer than 8% of participants (Supplementary Table S1). Baseline characteristics of the sample are presented in Table 1.

**TABLE 1:** Baseline characteristics.

Baseline variables	Mean ± SD (*n* %)
Age, years	59.2 ± 12.2; Range 40–97
40–64 years	922 (67.9)
≥ 65 years	434 (32)
Female	922 (68.0)
Education, years	9.8 (4.6)
Hispanic ethnicity	805 (59.9)
Race	
White	1,280 (96.3)
Black	49 (3.6)
Income	
≤ $20 000	593 (44.6)
>$20 000	738 (55.4)
Dementia/MCI	26 (1.9%)/214 (15.8%)
BMI, kg/m^2^	30.6 ± 6.4
Abdominal circumference, inches	38.7 ± 6.0
Systolic BP, mmHg	129.1 ± 17.6
HbA1c, %	6.2 ± 1.5
Fasting glucose, mg/dL	113.8 ± 47.7
HDL, mg/dL	50.0 ± 14.9
Triglycerides, mg/dL	160.2 ± 108.1
CRP, mg/L	2.7 ± 4.0
GGT, U/L	35.5 ± 55.5
EXIT-25	6.7 ± 4.4
TMT-A, s	53.8 ± 30.7
TMT-B, s	116.1 ± 63.8
RBANS attention	84.5 ± 20.9
RBANS language	91.2 ± 12.8
RBANS visuospatial/constructional	80.1 ± 16.3
RBANS immediate memory	90.8 ± 17.6
RBANS delayed recall	91.7 ± 15.1
CLOX	25.4 (3.1)

CRP, C-Reactive Protein; GGT, Gamma-glutamyl transferase; FBS, Fasting Blood Glucose; BMI, Body Mass Index; HDL, High-density lipoprotein; RBANS, Repeatable Battery for the Assessment of Neuropsychological Status; EXIT 25, Executive Interview 25; CLOX, Clock Drawing Test; TMT-A, Trail Making Test A; TMT-B, Trail Making Test B.

### Canonical correlation analysis: cognitive-biological associations

**FIGURE 1:**
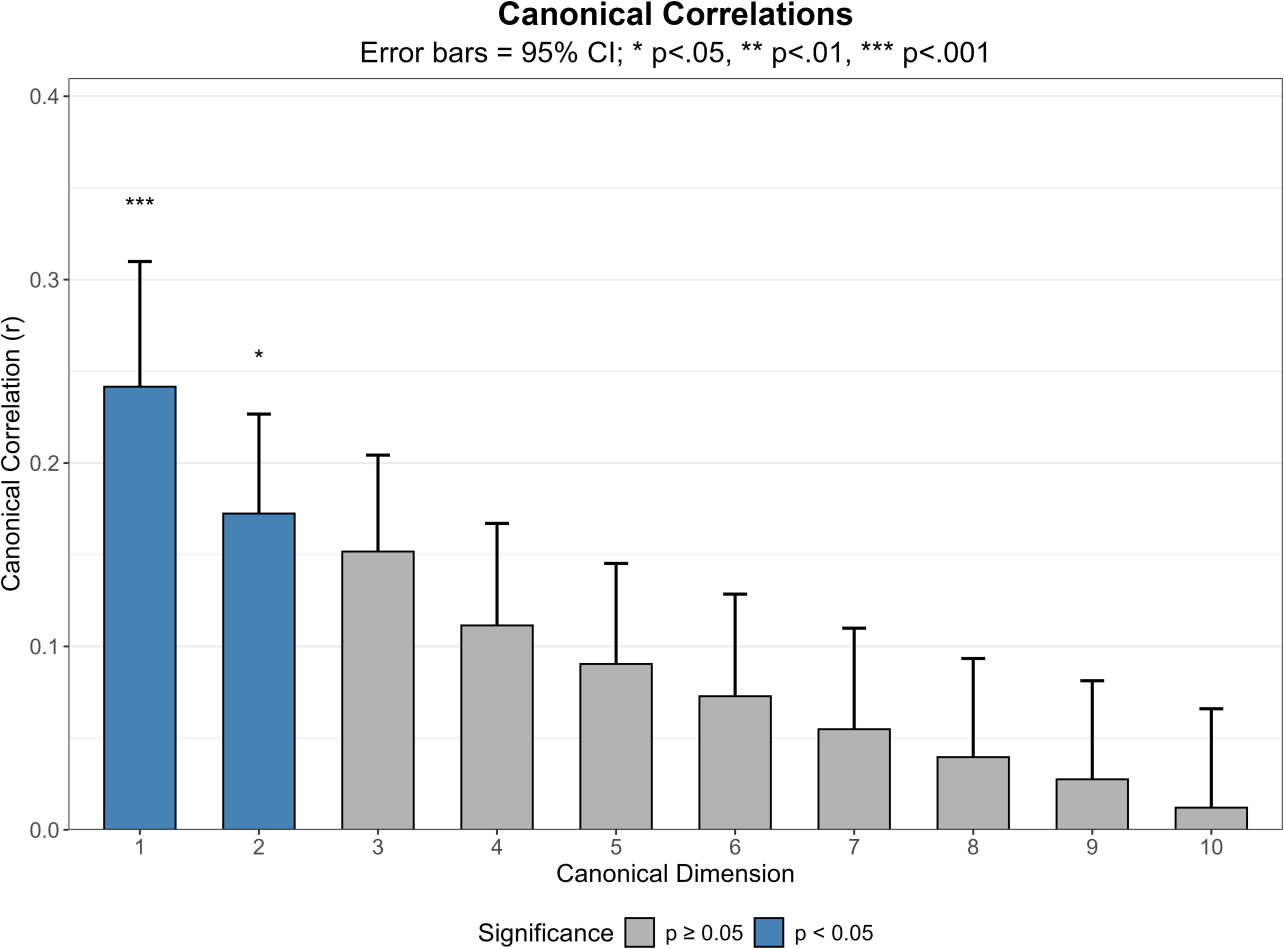
Canonical correlations across dimensions. bar heights represent the canonical correlation coefficients for each dimension, with error bars indicating 95% confidence intervals derived from Fisher’s z-transformation across 20 multiply imputed datasets. Asterisks denote statistical significance based on Wilks’ Λ tests (*p < 0.05, **p < 0.01, ***p < 0.001).

CCA revealed a significant multivariate association between cognitive performance and inflammatory-metabolic biomarkers. The first canonical correlation was *r* = 0.242 (95% CI: 0.171–0.310), accounting for 5.8% of the shared variance between the cognitive and biological domains (Figure 1 and Table 2). This association was statistically significant based on both Wilks’ Λ (*p* = 1.05 × 10^–6^) and permutation testing (Fisher combined *p* < 0.001). The canonical correlation was robust to bootstrap validation (*r* = 0.255, 95% CI: 0.219–0.294, 5,000 bootstrap samples), closely aligning with the pooled estimate from multiply imputed data and confirming the stability of the observed association between biological and cognitive variable sets. Canonical weights estimated in training folds were applied to held-out folds in five-fold cross-validation. The first canonical correlation decreased from r_1_ = 0.33 in-sample to r_1_ = 0.18 out-of-sample, suggesting some optimism in the data-driven procedure. However, the persistence of a positive held-out association suggests that the leading multivariate pattern was not solely an artifact of overfitting.

**TABLE 2:** Canonical correlation analysis summary.

Statistic	Value
First canonical correlation (r_1_)	0.242
95% Confidence interval	[0.171, 0.310]
Shared variance (r_1_^2^)	5.8%
Wilks’ Lambda *p*-value	1.05 × 10^–6^
Permutation *p*-value (Fisher combined)	<0.001
Number of Imputations	20
Permutations per Imputation	1,000

**FIGURE 2:**
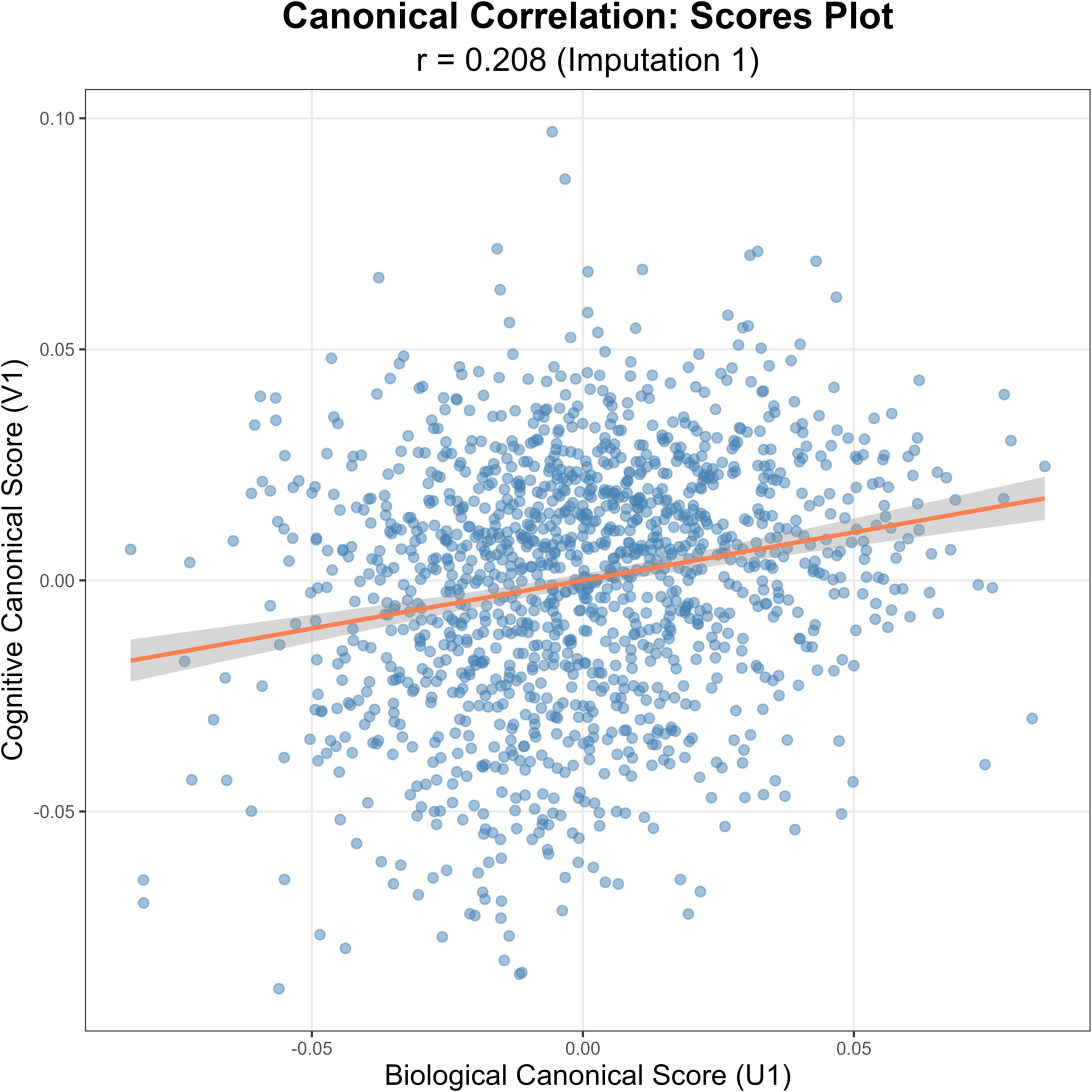
Scatter plot of canonical scores. Each point represents an individual participant’s scores on the cognitive (U_1_) and biological (V_1_) canonical variates derived from the first canonical dimension. The dashed line indicates the linear regression fit (*r* = 0.24, *p* < 0.001). Higher biological variate scores reflect greater inflammatory/metabolic burden; higher cognitive variate scores reflect poorer cognitive performance. Notably, participants in the upper-right quadrant exhibited both elevated biological risk and cognitive impairment, while those in the lower-left quadrant demonstrated favorable profiles on both dimensions. Scores were computed from the first multiply imputed dataset after covariate adjustment.

The canonical scores scatter plot (Figure 2) displays individual participant scores on the biological (U_1_) and cognitive (V_1_) canonical variates. The positive linear relationship indicates that individuals with higher biological variate scores (reflecting greater inflammatory and metabolic burden) tended to have higher cognitive variate scores (reflecting poorer cognitive performance).

#### Biological canonical loadings

**FIGURE 3:**
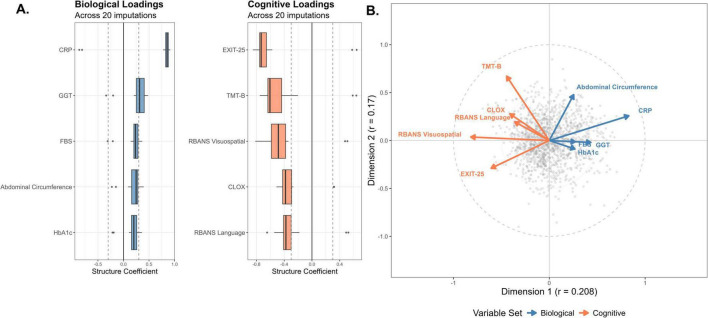
**(A)** Structure loadings across multiply imputed datasets. Boxplots show variability in structure loadings across 20 imputations for cognitive (left) and biological (right) variables. Dashed lines indicate | *r*| = 0.30 threshold. C-reactive protein and executive function measures (EXIT-25, TMT-B) demonstrated the strongest and most stable loadings. **(B)** Canonical Correlation Analysis Biplot of Cognitive and Biological Variables. Structure loadings on canonical dimensions 1 (*r* = 0.208) and 2 (*r* = 0.170). The opposing positions of cognitive (left) and biological (right) variables on Dimension 1 indicate that elevated inflammatory/metabolic markers are associated with poorer cognitive performance, particularly executive function. Loadings pooled across 20 imputed datasets, adjusted for demographics.

Inflammatory and metabolic markers predominantly characterized the biological canonical variate. CRP showed the strongest loading (0.69), followed by GGT (0.28), FBS (0.21), abdominal circumference 0.20), and hemoglobin A1c (0.18). All top biological loadings demonstrated high sign stability (90%) (Figure 3A and Table 3). The positive loadings indicate that the biological canonical variate represents a dimension of elevated inflammatory and metabolic burden.

**TABLE 3:** Canonical correlation analysis: cognitive and biological variable loadings.

Variable	Domain	Loading	Stability
(A) Biological variables			
CRP	Inflammation	0.69	0.90
GGT	Liver/inflammation	0.28	0.90
FBS	Glycemic	0.21	0.90
Abdominal circumference	Anthropometric	0.20	0.90
Hemoglobin A1c	Glycemic	0.18	0.90
Albumin	Nutritional	−0.04	0.70
BMI	Anthropometric	0.04	0.60
HDL	Lipid	0.03	0.65
Creatinine	Renal function	0.02	0.65
Triglycerides	Lipid	0.02	0.65
(B) Cognitive variables			
EXIT-25	Executive function	−0.59	0.90
Trail making test B	Executive function	−0.45	0.90
RBANS visuospatial	Visuospatial/constructional	−0.41	0.90
CLOX	Executive function	−0.31	0.90
RBANS language	Language	−0.30	0.90
FAS verbal fluency	Language	−0.19	0.90
RBANS delayed memory	Memory	−0.18	0.85
RBANS attention	Attention	−0.15	0.85
Trail making test A	Processing speed	−0.13	0.75
RBANS immediate memory	Memory	−0.12	1.00

CRP, C-Reactive Protein; GGT, Gamma-glutamyl transferase; FBS, Fasting Blood Glucose; BMI, Body Mass Index; HDL, High-density lipoprotein; RBANS, Repeatable Battery for the Assessment of Neuropsychological Status; EXIT 25, Executive Interview 25; CLOX, Clock Drawing Test; TMT-A, Trail Making Test A; TMT-B, Trail Making Test B; Loadings represent structure coefficients (correlations between original variables and canonical variates) pooled across 20 multiply imputed datasets. Sign stability indicates the proportion of imputations in which the loading sign matched the pooled estimate. Variables are ordered by absolute loading magnitude within each panel. Skewed variables (creatinine, CRP, GGT, triglycerides, TMT-A, TMT-B, EXIT-25, BMI, abdominal circumference) were log-transformed prior to analysis. To ensure consistent directionality, where higher scores indicate better cognitive performance, TMT-A and B and EXIT-25 scores were reverse-coded.

#### Cognitive canonical loadings

The cognitive canonical variate was most strongly defined by executive function. EXIT-25 (loading = −0.59) showed the strongest contribution, followed by Trail Making Test Part B (loading = −0.45), visuospatial/constructional abilities (RBANS-Visuospatial; loading = −0.41), clock drawing (CLOX; loading = −0.31), and language (RBANS-Language; loading = −0.30). All top cognitive loadings demonstrated high sign stability across imputations (90%), indicating robust, replicable patterns. The negative loadings indicate that the cognitive canonical variate represents a dimension where higher scores reflect poorer cognitive performance.

The CCA biplot (Figure 3B) illustrates the structure loadings of cognitive and biological variables on the first two canonical dimensions. Notably, cognitive and biological variables loaded on opposite sides of Dimension 1, with cognitive measures clustering in the negative direction and biological markers in the positive direction. The opposing orientation of these variable clusters indicates that elevated inflammatory and metabolic burden is associated with poorer cognitive performance, with executive dysfunction showing the strongest relationship. Dimension 2 (*r* = 0.170) captured additional variance, with modest differentiation between memory-related cognitive measures and adiposity-related biological markers, though this dimension contributed less to the overall multivariate association.

Redundancy analysis indicated that biomarkers explained approximately 1.0% of variance in the cognitive variable set on the first canonical dimension, and 1.34% cumulatively across the first two dimensions (1.82% cumulatively across all dimensions, Supplementary Figure S1). Permutation testing indicated that redundancy for the leading dimensions exceeded that expected under the null (*p* < 0.001).

#### Sensitivity analysis

There were 26 participants (1.9%) who carried a dementia classification, and 214 (15.8%) met criteria for mild cognitive impairment. A sensitivity analysis excluding these participants (analytic *n* = 1,117) yielded an essentially unchanged first canonical correlation (r_1_ = 0.226; 95% CI: 0.158–0.291). Furthermore, a sensitivity analysis that additionally adjusted for county of residence yielded a first canonical correlation that was materially unchanged (r_1_ = 0.209; 95% CI: 0.150–0.265). Moreover, in an exploratory income-stratified analysis, the first canonical correlation was 0.328 (95% CI: 0.252–0.405) in the higher-income and 0.282 (95% CI: 0.187–0.352) in the lower-income subgroup, indicating a comparable association across income strata.

#### Structural equation modeling

**FIGURE 4:**
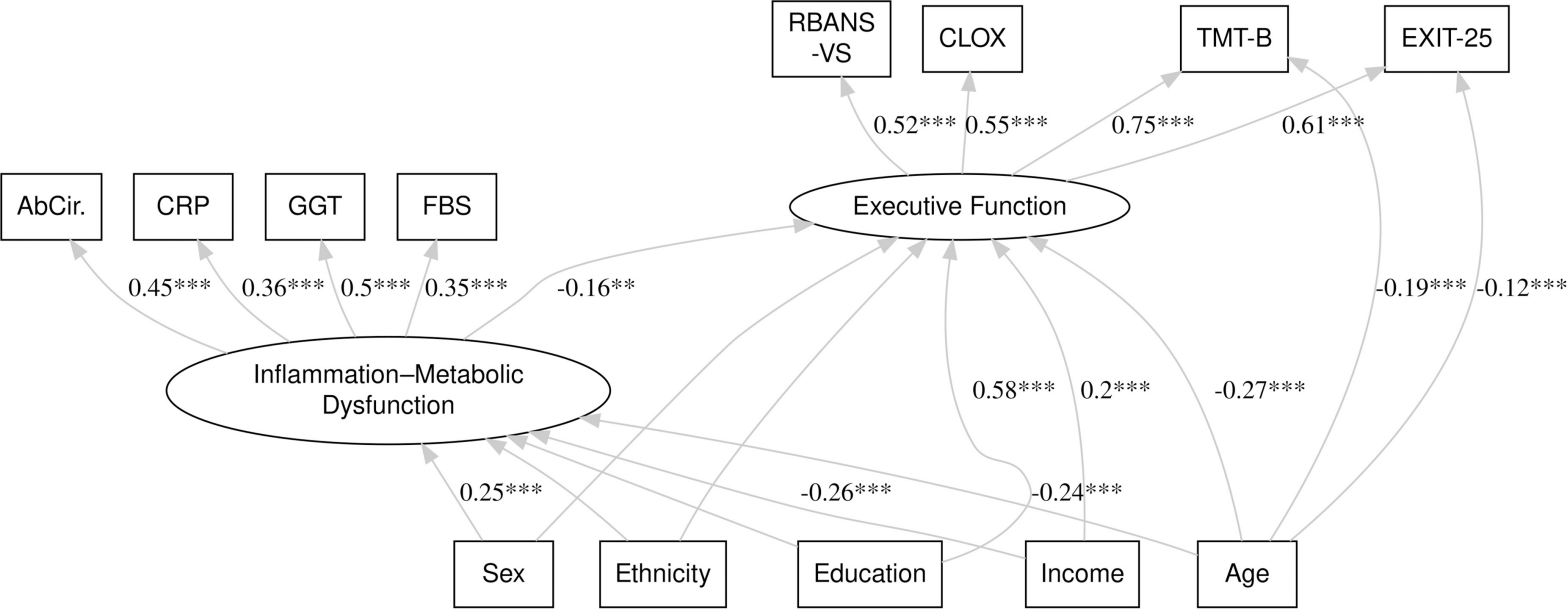
Path diagram for the structural equation model of the association between inflammation, metabolic dysfunction, and executive function. Latent variables are shown as ovals and observed variables as rectangles. The latent inflammation-metabolic dysfunction construct was measured by C-reactive protein (CRP), gamma-glutamyl transferase (GGT), fasting blood sugar (FBS), and abdominal circumference (AbCir). The latent executive function construct was measured using the Executive Interview-25 (EXIT-25), the Trail Making Test Part B (TMT-B), the RBANS Visuospatial/Constructional (RBANS-VS), and the Clock Drawing Test (CLOX). Two residual covariances were retained between RBANS-VS and CLOX, and between FBS and AbCir. Higher inflammation-metabolic dysfunction was significantly associated with lower executive function, controlling for sex, ethnicity, education, income, and age. Numbers next to arrows represent standardized coefficients (*p < 0.05, **p < 0.01, ***p < 0.001). The diagram was created from the first multiply imputed dataset after covariate adjustment.

In the pooled multiple-imputation SEM (20 imputations; robust ML), overall fit was acceptable (χ^2^ = 204.1, df = 45, *p* < 0.001; CFI = 0.929; TLI = 0.893; RMSEA = 0.051, 90% CI 0.044–0.058; SRMR = 0.040). TLI was marginally below the conventional threshold of 0.9 (Hu and Bentler, 1999). TLI is a parsimony-penalizing, non-normed index that is biased downward when between-set associations are modest (Hu and Bentler, 1999), we interpret the converging evidence from the CFI, RMSEA, and SRMR as indicating acceptable global fit. Higher inflammatory-metabolic burden was associated with worse executive function (β = −0.310, SE = 0.086, df = 507.28, *p* < 0.001; standardized β = −0.202), controlling for age, sex, ethnicity, education, and income (Figure 4 and Supplementary Table S2).

## Discussion

In this study, we employed CCA to elucidate the complex, multivariate relationship between systemic inflammatory-metabolic markers and cognitive performance in a large, rural community-based cohort of middle-aged and older adults in the West Texas region. The analysis revealed that a biological profile characterized by heightened inflammation and metabolic dysregulation was associated with a cognitive profile marked by diminished executive function. Notably, this association remained significant after adjustment for age, sex, ethnicity, education, and income. Hence, our findings provide multivariate, data-driven support for the hypothesis that systemic health is linked to cognitive performance in later life (Chrzanowski et al., 2025).

Our principal finding was a significant first canonical correlation, which, while modest in magnitude, underscores the complex and multifactorial nature of cognitive aging. The biological canonical variate was most heavily weighted by CRP, a well-established marker of systemic inflammation (Bettcher et al., 2012), with additional contributions from markers of metabolic and oxidative stress [i.e., GGT, (Praetorius Björk and Johansson, 2018) FBS, HbA1c (Chi et al., 2023)] and central adiposity (Norris et al., 2023). This pattern suggests the variate represents a latent dimension of cardiometabolic and inflammatory burden. Conversely, the cognitive canonical variate was predominantly defined by measures of executive functions, including the EXIT-25 and TMT-B. The opposing signs of the canonical loadings clearly indicate that a higher burden of systemic inflammation and metabolic dysregulation was associated with poorer executive performance. Our finding aligns with a substantial body of literature demonstrating the particular vulnerability of frontal-subcortical circuits, which subserve executive functions, to vascular and metabolic insults (Bettcher et al., 2012; Kipinoinen et al., 2022).

Furthermore, the emergence of CRP as the leading biological contributor is consistent with numerous studies linking it to cognitive decline and dementia risk (Long et al., 2023). Population-based studies have consistently shown that higher circulating CRP is associated with worse performance in cognitive domains such as executive function and memory, as well as an increased risk for incident dementia, including both Alzheimer’s disease and vascular dementia (Chen et al., 2019; Long et al., 2023; Tegeler et al., 2016; Warren et al., 2018; Wersching et al., 2010). However, several meta-analyses have provided inconsistent results due to significant heterogeneity between studies (Long et al., 2023; Yang et al., 2015). Our results extend these univariate findings by demonstrating that CRP’s influence on cognition occurs within a broader context of co-occurring metabolic disturbances. By using CCA, we move beyond one-to-one associations to characterize a holistic biological risk profile.

The pattern observed here suggests that a common pathway, likely involving vascular mechanisms, may connect systemic inflammation and metabolic dysregulation to cognitive impairment (Gorelick et al., 2011). Chronic inflammation promotes atherosclerosis and endothelial dysfunction, compromising cerebral blood flow and the integrity of the brain’s white matter, which in turn disrupts the distributed neural networks essential for cognition, including visuospatial and higher-order executive functions (Long et al., 2023; Wersching et al., 2010). For instance, Warren et al. (2018) showed that long-term elevated inflammatory markers such as CRP are associated with decreased cerebral blood flow in a wide range of areas involved in attention, executive function, and memory, including the medial temporal lobe and cingulate cortex. Nevertheless, this study itself did not find any significant changes in cognitive functions except for CLOX. Earlier reports from Yaffe et al. (2004) indicated that there is a higher likelihood of developing cognitive impairment with metabolic syndrome and increased inflammation (RR 1.66, 95% CI: 1.19–2.32). Here, the increased risk associated with the metabolic syndrome is modified by inflammation.

Interestingly, we have seen a prominent contribution from the GGT, which is not only considered an indicator of liver injury but also an early marker of oxidative stress (Praetorius Björk and Johansson, 2018). There is a growing body of evidence that suggests that elevated GGT is related to cognitive decline and dementia (Praetorius Björk and Johansson, 2018; Zhang et al., 2024). For instance, Kunutsor and Laukkanen (2016) showed that the hazard ratio for dementia per 1 standard deviation increase in baseline log GGT was 1.33 (95% CI: 1.14–1.55). Furthermore, Praetorius Björk and Johansson (2018) showed that higher GGT levels were associated with steeper cognitive decline in later life. A recent Mendelian randomization study showed that there is strong evidence that elevated GGT in mid-life is associated with cardiovascular injury and vascular dementia (Chen J. et al., 2024). Since our cohort comprises relatively middle-aged individuals, that finding is worth mentioning.

Similarly, our other prominent biomarkers, including short-term and long-term glycemic control and central obesity, have previously shown strong associations with cognitive impairment (Zheng et al., 2018). These cardiometabolic risk factors are thought to compromise brain health through various mechanisms, including cerebrovascular damage, impaired glucose metabolism, and oxidative stress, with executive functions appearing particularly vulnerable to this type of systemic biological insult. Interestingly, in our cohort, lipid parameters showed very weak loadings, similar to the previous literature (Koch et al., 2020; van den Kommer et al., 2012). Accordingly, the majority of previously published population studies provide inconclusive evidence regarding the association between HDL, triglycerides, and cognitive decline (Bates et al., 2017; Li et al., 2020; Xie et al., 2025). Similarly, SBP showed weaker loading, in accord with inconsistencies in the literature regarding cognitive functions (Williamson et al., 2019). Another possible reason is that most of the biomarkers have non-linear relationships, and by doing CCP analysis, we are working on linear combinations.

The findings of this study have important clinical and public health implications, particularly for the underserved rural population from which our sample was drawn. Given the modest shared variance, these associations are best regarded as hypothesis-generating: they identify a multivariate inflammatory-metabolic pattern that varies with executive performance, and that warrants prospective evaluation before any screening application is considered. Lifestyle modifications targeting diet, physical activity, and management of vascular risk factors are known to improve both metabolic health and inflammatory status and may represent a critical strategy for preserving cognitive function in aging populations (Ngandu et al., 2015) For rural communities, which often face a higher prevalence of cardiometabolic disease and limited access to healthcare, these findings highlight the urgent need for targeted public health initiatives aimed at promoting brain health through the management of systemic disease.

This study has several notable strengths, including its large, community-based sample, comprehensive assessment of both biological and cognitive domains, and the application of a sophisticated multivariate statistical approach. The use of CCA, combined with rigorous methods for handling missing data and validating results through permutation testing, provides a robust framework for interpreting these complex associations.

However, certain limitations must be acknowledged. First, the cross-sectional design precludes any inference of causality; we can identify an association but cannot determine if the biological state precedes and causes cognitive decline. Longitudinal follow-up is necessary to establish temporal precedence. Second, our CCA showed only a shared variance of 5%, lower than the 25% reported in a previous CCA study of small vessel disease (van Dinther et al., 2024). However, the previous study was targeting the neurovascular system *per se*. Not the whole metabolic profile. Since our study was based on broader biological variables, these results were expected. Furthermore, our variable set is not immune to measurement error, nonlinearity, and skewness. CCA assumes linear relationships and is not robust against nonlinearity. Furthermore, these results suggest that a substantial portion of the variance in cognitive performance is explained by factors not included in our model, such as genetics, lifestyle, and psychosocial variables. Moreover, the SEM indicator selection was guided by CCA loadings from the same analytic sample, potentially introducing circularity and overfitting. Although repeated cross-validation demonstrated a positive held-out canonical correlation, external validation in an independent cohort is needed before the latent structure can be interpreted as confirmatory. Third, while focusing on a rural cohort is a strength for addressing health disparities, it may also limit the generalizability of our findings to other populations with different demographic and socioeconomic characteristics. Finally, this sample predominantly included middle-aged individuals. As a result, the RBANS may be relatively easy for middle-aged adults, raising the possibility of ceiling effects in some domains; this could attenuate associations involving the RBANS indices and limit their sensitivity in a predominantly middle-aged sample. Accordingly, the cognitive battery is weighted toward executive and processing-speed measures (EXIT-25, TMT-A and B, FAS, CLOX, and RBANS Attention); the prominence of executive function in the cognitive canonical variate should therefore be interpreted in light of this composition. Given the strong loading of the RBANS-Visuospatial (e.g., Figure Copy vs. Line Orientation), decomposing it could clarify whether construction/planning demands drive its association with inflammatory-metabolic burden. Utilizing individual variables will be considered in our future studies.

Future research should build upon these findings through longitudinal analysis to track the co-evolution of biological and cognitive changes over time. Integrating neuroimaging data would be a valuable next step to elucidate the specific structural and functional brain changes that mediate the observed associations. Furthermore, intervention studies are needed to determine whether modifying the identified profile of inflammatory and metabolic risk can slow the trajectory of cognitive decline. In conclusion, this study provides robust, multivariate evidence linking a profile of systemic inflammation and metabolic dysregulation to executive dysfunction in a rural aging cohort. It reinforces the critical importance of maintaining cardiometabolic health as a cornerstone of preserving cognitive vitality in later life.

## Data Availability

The data supporting this study were collected through Project FRONTIER and are housed at the Garrison Institute on Aging at TTUHSC. Because the data contain potentially identifying information from a small rural cohort, they are not publicly available but may be obtained from the corresponding author upon reasonable request, provided appropriate institutional and ethical approvals are in place.

## References

[B1] Appiah D. Ashworth G. Boles A. Nair N. (2022). The association of heart/vascular aging with mild cognitive impairment in a rural multiethnic cohort: The project FRONTIER study. *J. Prev. Alzheimers Dis.* 9 315–322. 10.14283/jpad.2022.15 35543005

[B2] Army Individual Test Battery (1944). *Manual of Directions and Scoring.* Washington, DC: War Department, Adjutant General’s Office.

[B3] Ashendorf L. Jefferson A. L. O’Connor M. K. Chaisson C. Green R. C. Stern R. A. (2008). Trail Making Test errors in normal aging, mild cognitive impairment, and dementia. *Arch. Clin. Neuropsychol.* 23 129–137. 10.1016/j.acn.2007.11.005 18178372 PMC2693196

[B4] Bates K. A. Sohrabi H. R. Rainey-Smith S. R. Weinborn M. Bucks R. S. Rodrigues M. et al. (2017). Serum high-density lipoprotein is associated with better cognitive function in a cross-sectional study of aging women. *Int. J. Neurosci.* 127 243–252. 10.1080/00207454.2016.1182527 27113638

[B5] Bettcher B. M. Wilheim R. Rigby T. Green R. Miller J. W. Racine C. A. et al. (2012). C-reactive protein is related to memory and medial temporal brain volume in older adults. *Brain Behav. Immun.* 26 103–108. 10.1016/j.bbi.2011.07.240 21843630 PMC3221922

[B6] Borkowski J. G. Benton A. L. Spreen O. (1967). Word fluency and brain damage. *Neuropsychologia* 5 135–140. 10.1016/0028-3932(67)90015-2

[B7] Chen C. Liu Y. Cao Z. Yin Z. Zhao F. Lv Y. et al. (2019). Combined associations of hs-CRP and cognitive function with all-cause mortality among oldest-old adults in Chinese longevity areas: A prospective cohort study. *Immun. Ageing* 16:30. 10.1186/s12979-019-0170-y 31832073 PMC6859603

[B8] Chen J. Liang C. Liu C. Jie L. Liu B. Yang X. (2024). Liver enzyme and risk of vascular dementia: A univariable and multivariable Mendelian randomization of European descent. *Neuroprotection* 2 310–317. 10.1002/nep3.67 41383373 PMC12486898

[B9] Chen M. D. Deng C. F. Chen P. F. Li A. Wu H. Z. Ouyang F. et al. (2024). Non-invasive metabolic biomarkers in initial cognitive impairment in patients with diabetes: A systematic review and meta-analysis. *Diabetes Obes. Metab.* 26 5519–5536. 10.1111/dom.15916 39233493

[B10] Cheng Y. Shao C. Lathrop Q. N. (2016). The mediated MIMIC model for understanding the underlying mechanism of DIF. *Educ. Psychol. Meas.* 76 43–63. 10.1177/0013164415576187 29795856 PMC5965572

[B11] Chi H. Song M. Zhang J. Zhou J. Liu D. (2023). Relationship between acute glucose variability and cognitive decline in type 2 diabetes: A systematic review and meta-analysis. *PLoS One* 18:e0289782. 10.1371/journal.pone.0289782 37656693 PMC10473499

[B12] Chrzanowski L. Singer J. Rerick P. Elliott L. Levitt D. E. Cummings C. et al. (2025). The role of inflammation, chronic pain, and hypertension on cognitive functioning in an underserved, rural population: A Project FRONTIER study. *J. Clin. Exp. Neuropsychol.* 47 249–262. 10.1080/13803395.2025.2527341 40662212

[B13] Cui Y. Li C. Ke B. Xiao Y. Wang S. Jiang Q. et al. (2024). Protective role of serum albumin in dementia: A prospective study from United Kingdom biobank. *Front. Neurol.* 15:1458184. 10.3389/fneur.2024.1458184 39206288 PMC11349656

[B14] Dyer A. H. McNulty H. Caffrey A. Gordon S. Laird E. Hoey L. et al. (2024). Low-Grade systemic inflammation is associated with domain-specific cognitive performance and cognitive decline in older adults: Data from the TUDA study. *Neurobiol. Aging* 134 94–105. 10.1016/j.neurobiolaging.2023.11.008 38043161

[B15] Elias M. F. Elias P. K. Sullivan L. M. Wolf P. A. D’Agostino R. B. (2005). Obesity, diabetes and cognitive deficit: The Framingham Heart Study. *Neurobiol. Aging* 26 (Suppl. 1), 11–16. 10.1016/j.neurobiolaging.2005.08.019 16223549

[B16] Gasparini F. Valletta M. Vetrano D. L. Beridze G. Rizzuto D. Calderón-Larrañaga A. et al. (2026). Kidney function, Alzheimer disease blood biomarkers, and dementia risk in community-dwelling older adults. *Neurology* 106:e214446. 10.1212/wnl.0000000000214446 41337685 PMC12687484

[B17] Gorelick P. B. Scuteri A. Black S. E. Decarli C. Greenberg S. M. Iadecola C. et al. (2011). Vascular contributions to cognitive impairment and dementia: A statement for healthcare professionals from the American heart association/American stroke association. *Stroke* 42 2672–2713. 10.1161/STR.0b013e3182299496 21778438 PMC3778669

[B18] Han L. Li Q. Zhang L. Yu J. Liu Y. Li W. et al. (2025). The necessity of strengthening glycemic and lipid metabolism management for improving brain structure and cognitive function in people with diabetes: A retrospective study based on UK Biobank. *Diabetes Res. Clin. Pract.* 226:112366. 10.1016/j.diabres.2025.112366 40659220

[B19] Hotelling H. (1992). “Relations between two sets of variates,” in *Breakthroughs in Statistics: Methodology and Distribution*, eds Kotz S. Johnson N. L. (New York, NY: Springer), 162–190.

[B20] Hu L. T. Bentler P. M. (1999). Cutoff criteria for fit indexes in covariance structure analysis: Conventional criteria versus new alternatives. *Struct. Equ. Modeling* 6 1–55. 10.1080/10705519909540118

[B21] Huang Y. Y. Wang H. F. Wu B. S. Ou Y. N. Ma L. Z. Yang L. et al. (2024). Clinical laboratory tests and dementia incidence: A prospective cohort study. *J. Affect. Disord.* 351 1–7. 10.1016/j.jad.2024.01.226 38286224

[B22] Kałamała P. Ware N. Fabiani M. Michie P. Hunter M. Wade A. et al. (2025). Cardiorespiratory fitness and cardiometabolic health are associated with distinct cognitive domains in cognitively healthy older adults. *Sci. Rep.* 15:42849. 10.1038/s41598-025-26105-x 41315438 PMC12669785

[B23] Karpman M. B. (1981). Redundancy in canonical analysis. *Res. Q. Exerc. Sport* 52 291–292. 10.1080/02701367.1981.10607871

[B24] Kipinoinen T. Toppala S. Rinne J. O. Viitanen M. H. Jula A. M. Ekblad L. L. (2022). Association of midlife inflammatory markers with cognitive performance at 10-year follow-up. *Neurology* 99 e2294–e2302. 10.1212/wnl.0000000000201116 36195448 PMC9694835

[B25] Koch M. DeKosky S. T. Goodman M. Sun J. Furtado J. D. Fitzpatrick A. L. et al. (2020). High density lipoprotein and its apolipoprotein-defined subspecies and risk of dementia. *J. Lipid Res.* 61 445–454. 10.1194/jlr.P119000473 31892526 PMC7053836

[B26] Kunutsor S. K. Laukkanen J. A. (2016). Gamma glutamyltransferase and risk of future dementia in middle-aged to older Finnish men: A new prospective cohort study. *Alzheimers Dement.* 12 931–941. 10.1016/j.jalz.2016.03.003 27103259

[B27] Li J. Jiao M. Wen J. Fan D. Xia Y. Cao Y. et al. (2020). Association of body mass index and blood lipid profile with cognitive function in Chinese elderly population based on data from the China Health and Nutrition Survey, 2009-2015. *Psychogeriatrics* 20 663–672. 10.1111/psyg.12559 32339333

[B28] Lin Y. Gong Z. Ma C. Wang Z. Wang K. (2023). Relationship between glycemic control and cognitive impairment: A systematic review and meta-analysis. *Front. Aging Neurosci.* 15:1126183. 10.3389/fnagi.2023.1126183 36776436 PMC9909073

[B29] Long S. Chen Y. Meng Y. Yang Z. Wei M. Li T. et al. (2023). Peripheral high levels of CRP predict progression from normal cognition to dementia: A systematic review and meta-analysis. *J. Clin. Neurosci.* 107 54–63. 10.1016/j.jocn.2022.11.016 36502782

[B30] Miller J. B. Wong C. G. Caldwell J. Z. K. Rodrigues J. Pudumjee S. John S. E. et al. (2023). Cognitive aging in rural communities: Preliminary memory characterization of a community cohort from Southern Nevada. *Front. Dement.* 2:1236039. 10.3389/frdem.2023.1236039 39081981 PMC11285680

[B31] Ngandu T. Lehtisalo J. Solomon A. Levälahti E. Ahtiluoto S. Antikainen R. et al. (2015). A 2 year multidomain intervention of diet, exercise, cognitive training, and vascular risk monitoring versus control to prevent cognitive decline in at-risk elderly people (FINGER): A randomised controlled trial. *Lancet* 385 2255–2263. 10.1016/s0140-6736(15)60461-5 25771249

[B32] Norris T. Salzmann A. Henry A. Garfield V. Pinto Pereira S. M. (2023). The relationship between adiposity and cognitive function: A bidirectional Mendelian randomization study in UK Biobank. *Int. J. Epidemiol.* 52 1074–1085. 10.1093/ije/dyad043 37029912 PMC10396406

[B33] O’Bryant S. Zhang Y. Owen D. Cherry B. Silva M. Hudson C. et al. (2009). The Cochran county aging study: Methodology and descriptive statistics. *Tex. Public Health J.* 61 5–7. 10.1681/ASN.0000001007 41587095 PMC13337171

[B34] O’Bryant S. E. Falkowski J. Hobson V. Johnson L. Hall J. Schrimsher G. W. et al. (2011). Executive functioning mediates the link between other neuropsychological domains and daily functioning: A Project FRONTIER study. *Int. Psychogeriatr.* 23 107–113. 10.1017/s1041610210000967 20637139

[B35] Praetorius Björk M. Johansson B. (2018). Gamma-Glutamyltransferase (GGT) as a biomarker of cognitive decline at the end of life: Contrasting age and time to death trajectories. *Int. Psychogeriatr.* 30 981–990. 10.1017/s1041610217002393 29108523

[B36] Randolph C. Tierney M. C. Mohr E. Chase T. N. (1998). The Repeatable Battery for the Assessment of Neuropsychological Status (RBANS): Preliminary clinical validity. *J. Clin. Exp. Neuropsychol.* 20 310–319. 10.1076/jcen.20.3.310.823 9845158

[B37] Ravaglia G. Forti P. Maioli F. Chiappelli M. Montesi F. Tumini E. et al. (2007). Blood inflammatory markers and risk of dementia: The Conselice Study of Brain Aging. *Neurobiol. Aging* 28 1810–1820. 10.1016/j.neurobiolaging.2006.08.012 17011077

[B38] Reitan R. M. (1958). Validity of the trail making test as an indicator of organic brain damage. *Percept. Mot. Skills* 8 271–276. 10.2466/pms.1958.8.3.271

[B39] Royall D. R. Cordes J. A. Polk M. (1998). CLOX: An executive clock drawing task. *J. Neurol. Neurosurg. Psychiatry* 64 588–594. 10.1136/jnnp.64.5.588 9598672 PMC2170069

[B40] Royall D. R. Mahurin R. K. Gray K. F. (1992). Bedside assessment of executive cognitive impairment: The executive interview. *J. Am. Geriatr. Soc.* 40 1221–1226. 10.1111/j.1532-5415.1992.tb03646.x 1447438

[B41] Saczynski J. S. Jónsdóttir M. K. Garcia M. E. Jonsson P. V. Peila R. Eiriksdottir G. et al. (2008). Cognitive impairment: An increasingly important complication of type 2 diabetes: The age, gene/environment susceptibility–Reykjavik study. *Am. J. Epidemiol.* 168 1132–1139. 10.1093/aje/kwn228 18836152 PMC2727243

[B42] Sartori A. C. Vance D. E. Slater L. Z. Crowe M. (2012). The impact of inflammation on cognitive function in older adults: Implications for healthcare practice and research. *J. Neurosci. Nurs.* 44 206–217. 10.1097/JNN.0b013e3182527690 22743812 PMC3390758

[B43] Solomon A. Kåreholt I. Ngandu T. Wolozin B. Macdonald S. W. Winblad B. et al. (2009a). Serum total cholesterol, statins and cognition in non-demented elderly. *Neurobiol. Aging* 30 1006–1009. 10.1016/j.neurobiolaging.2007.09.012 18022292

[B44] Solomon A. Kivipelto M. Wolozin B. Zhou J. Whitmer R. A. (2009b). Midlife serum cholesterol and increased risk of Alzheimer’s and vascular dementia three decades later. *Dement. Geriatr. Cogn. Disord.* 28 75–80. 10.1159/000231980 19648749 PMC2814023

[B45] Tegeler C. O’Sullivan J. L. Bucholtz N. Goldeck D. Pawelec G. Steinhagen-Thiessen E. et al. (2016). The inflammatory markers CRP, IL-6, and IL-10 are associated with cognitive function–data from the Berlin Aging Study II. *Neurobiol. Aging* 38 112–117. 10.1016/j.neurobiolaging.2015.10.039 26827649

[B46] Van Buuren S. Groothuis-Oudshoorn K. (2011). mice: Multivariate imputation by chained equations in R. *J. Stat. Softw.* 45 1–67. 10.18637/jss.v045.i03

[B47] van den Kommer T. N. Dik M. G. Comijs H. C. Jonker C. Deeg D. J. (2012). Role of lipoproteins and inflammation in cognitive decline: Do they interact? *Neurobiol. Aging* 33 196.e1–196.e12. 10.1016/j.neurobiolaging.2010.05.024 20594617

[B48] van Dinther M. Voorter P. H. M. Zhang E. van Kuijk S. M. J. Jansen J. F. A. van Oostenbrugge R. J. et al. (2024). The neurovascular unit and its correlation with cognitive performance in patients with cerebral small vessel disease: A canonical correlation analysis approach. *Geroscience* 46 5061–5073. 10.1007/s11357-024-01235-8 38888875 PMC11335703

[B49] Warren K. N. Beason-Held L. L. Carlson O. Egan J. M. An Y. Doshi J. et al. (2018). Elevated markers of inflammation are associated with longitudinal changes in brain function in older adults. *J. Gerontol. A Biol. Sci. Med. Sci.* 73 770–778. 10.1093/gerona/glx199 29304217 PMC5946946

[B50] Wersching H. Duning T. Lohmann H. Mohammadi S. Stehling C. Fobker M. et al. (2010). Serum C-reactive protein is linked to cerebral microstructural integrity and cognitive function. *Neurology* 74 1022–1029. 10.1212/WNL.0b013e3181d7b45b 20350977

[B51] Williamson J. D. Pajewski N. M. Auchus A. P. Bryan R. N. Chelune G. Cheung A. K. et al. (2019). Effect of intensive vs standard blood pressure control on probable dementia: A randomized clinical trial. *JAMA* 321 553–561. 10.1001/jama.2018.21442 30688979 PMC6439590

[B52] Xie L. Luo H. Zhao Y. Hao Y. Gao J. Sun C. et al. (2025). Triglycerides, high-density lipoprotein and cognitive function in middle-aged and older adults: A cross-sectional analysis. *Biogerontology* 26:75. 10.1007/s10522-025-10201-6 40119954

[B53] Yaffe K. Kanaya A. Lindquist K. Simonsick E. M. Harris T. Shorr R. I. et al. (2004). The metabolic syndrome, inflammation, and risk of cognitive decline. *JAMA* 292 2237–2242. 10.1001/jama.292.18.2237 15536110

[B54] Yang J. Fan C. Pan L. Xie M. He Q. Li D. et al. (2015). C-reactive protein plays a marginal role in cognitive decline: A systematic review and meta-analysis. *Int. J. Geriatr. Psychiatry* 30 156–165. 10.1002/gps.4236 25475551

[B55] Zhang Y. L. Jia S. Y. Yang B. Miao J. Su C. Cui Z. G. et al. (2024). Non-linear association of liver enzymes with cognitive performance in the elderly: A cross-sectional study. *PLoS One* 19:e0306839. 10.1371/journal.pone.0306839 39042647 PMC11265699

[B56] Zheng F. Yan L. Yang Z. Zhong B. Xie W. (2018). HbA1c, diabetes and cognitive decline: The English Longitudinal Study of Ageing. *Diabetologia* 61 839–848. 10.1007/s00125-017-4541-7 29368156 PMC6448974

[B57] Zhong Y. Li L. (2025). Association between liver biomarkers and risk of cognitive impairment and dementia: A systematic review and meta-analysis. *Pak. J. Med. Sci.* 41 2122–2132. 10.12669/pjms.41.7.12321 40735554 PMC12302112

