# Supplementary material for: Multivariate associations between systemic inflammation, metabolic dysregulation, and cognitive performance in project FRONTIER
Source: Front Aging Neurosci. 2026 Jul 14;18:1836968. doi: 10.3389/fnagi.2026.1836968 (PMC13407628; doi:10.3389/fnagi.2026.1836968)
Supplement: Supplementary file 1 [file Data_Sheet_1.docx]

**SUPPLEMENTARY MATERIAL**

**Multivariate Associations Between Systemic Inflammation, Metabolic Dysregulation, and Cognitive Performance in Project FRONTIER**

**SUPPLEMENTARY METHODS**

**Statistical Analysis**

*Canonical Correlation Analysis (CCA)*

CCA provides a comprehensive framework for examining interdependencies between variable groups and for reducing data dimensionality. CCA was first introduced by Harold Hotelling in 1936 as a technique for identifying linear combinations, called canonical variates, from two multivariate datasets such that the correlation between these combinations is maximized. ^1^ In other words, it determines the directions in which the variables in one set are most strongly associated with the variables in the other set. Once the first pair of maximally correlated canonical variates is obtained, subsequent pairs are extracted under the constraint that they are uncorrelated with all previously derived pairs, ensuring that each new pair captures a unique aspect of the relationship. This sequence of uncorrelated variates efficiently summarizes the association structure between the two datasets and interprets their joint variability with fewer dimensions. Therefore, CCA was employed to examine the multivariate relationship between cognitive performance and biological markers. CCA identifies linear combinations of variables within each set (canonical variates) that maximize the correlation between sets, providing a comprehensive assessment of shared variance between cognitive and biological domains.

*Variable Sets*

The cognitive variable set included measures of memory ^2^ (RBANS-IM and RBANS-DM), attention (RBANS-Attention), language (RBANS-Language, FAS verbal fluency^3^), visuospatial/constructional abilities (RBANS-Visuospatial), and executive function (EXIT-25, ^4^ TMT-B, ^5-7^ CLOX ^8^), and processing speed (TMT-A) ^5-7^. A domain-driven metabolic marker set was selected to examine the associations between systematic metabolic health and cognitive performance. This metabolic marker set comprised indices of chronic and short-term glycemic control (haemoglobin A1c-HbA1c, fasting blood glucose-FBS), ^9, 10^ lipid metabolism (triglycerides, high density lipoprotein-HDL), ^11, 12^ nutritional status and adiposity (albumin, ^13^ body mass index-BMI, ^14, 15^, abdominal circumference ^16^), hemodynamic load (systolic blood pressure-SBP ^17, 18^), systemic inflammation (C-reactive protein-CRP ^19-21^), renal function (creatinine ^22^), and hepatic/metabolic stress (gamma-glutamyl transferase, GGT). ^23, 24^ These markers were selected because each has been robustly linked to cognitive impairment, dementia risk, or relevant cerebrovascular/structural brain changes in epidemiologic and clinical cohorts, and together they capture complementary dimensions of cardiometabolic risk, inflammation, and end-organ damage that plausibly influence late-life cognition via vascular, neurodegenerative, and oxidative stress mechanisms. ^24-27^

*Handling of Missing Data*

Multiple imputation was used to address missing data under the missing-at-random (MAR) assumption. Twenty imputed datasets were generated using the “mice” package in R. ^28^ Predictive mean matching (PMM) was used for continuous variables, while classification and regression trees (CART) were employed for categorical variables to avoid separation issues inherent in logistic regression with sparse categorical data. Predictor selection was performed using the quickpred algorithm, with a minimum correlation of 0.05 and a minimum usable-case proportion of 0.25 to reduce collinearity.

*Variable Transformation*

Prior to imputation, distributional properties of all continuous variables were examined using skewness statistics. Variables exhibiting substantial right-skewness (skewness > 1.0) were log-transformed to improve normality and linearity of relationships. Specifically, natural log transformations were applied to creatinine, CRP, GGT, triglycerides, TMT A and B, EXIT-25, BMI, and abdominal circumference. For variables with zero or near-zero values, log(1 + x) transformation was used. Fasting glucose was winsorized at the 1st and 99th percentiles to address extreme outliers while preserving the bulk of the distribution. To ensure consistent directionality, where higher scores indicate better cognitive performance (TMT-A and B; higher times indicate worse performance) and higher scores indicate worse executive function (EXIT-25), TMT-A and B and EXIT-25 scores were reverse-coded during the CCA analysis.

*Covariate Adjustment*

Prior to CCA, all cognitive and biological variables were residualized on demographic covariates (age, sex, education, income, and ethnicity) using linear regression. This approach removes confounding effects while preserving the multivariate structure necessary for CCA.

*Pooling Across Imputations*

CCA was performed separately on each imputed dataset. Canonical correlations were pooled using Fisher's z-transformation with Rubin's rules to obtain combined estimates and 95% confidence intervals. Structure loadings (correlations between original variables and canonical variates) were averaged across imputations. To address potential sign indeterminacy of canonical variates across imputations, loadings were aligned to ensure consistent directionality before averaging. Sign stability was calculated as the proportion of imputations in which each loading of each variable matched the sign of the pooled loading. Statistical significance was assessed using two complementary approaches. First, Wilks' Lambda (Λ) with asymptotic F-approximation was computed for each imputed dataset, and median p-values were reported. Second, permutation testing (5,000 permutations per imputation) was conducted, with p-values combined across imputations using Fisher's method. To evaluate the internal reproducibility of the leading CCA dimension, we performed repeated 5-fold cross-validation of the canonical correlation for completed data sets.

*Redundancy Analysis and Permutation Testing*

Redundancy analysis was performed to quantify the proportion of variance in each variable set explained by the canonical variates of the opposite set. ^29^ For each canonical dimension d, the redundancy index was computed as the product of the average squared structure loading (i.e., the proportion of variance in a given set extracted by its own canonical variate) and the squared canonical correlation, yielding the proportion of variance in one set accounted for by the other set's canonical variate. Total redundancy was obtained by summing dimension-specific indices across all canonical dimensions, and the Stewart-Love index was calculated as the sum of bidirectional total redundancy indices to characterize the overall shared variance between the two variable sets.

Statistical significance of the redundancy indices was evaluated using permutation testing. For each multiply imputed dataset, 1,000 permutations were conducted by randomly shuffling the rows of the cognitive variable matrix while holding the biological variable matrix fixed, thereby disrupting the association between sets while preserving the within-set correlation structure. For each permutation, a full canonical correlation analysis was performed, and the redundancy indices were recomputed, generating empirical null distributions for both dimension-specific and total redundancy indices. One-tailed permutation p-values were calculated as the proportion of permuted values equal to or exceeding the observed redundancy index. Permutation p-values across the 20 multiply imputed datasets were combined using Fisher's method. All analyses were conducted in R version 4.5.2 using the mice, CCA, and CCP packages. Statistical significance was set at α = 0.05.

*Structural equation model*

We selected indicators based on the largest absolute structure coefficients on the primary canonical dimension (the first canonical function), prioritizing variables that were consistently strong contributors across imputations. The CCA-informed indicators were then carried forward to the SEM as observed indicators of two latent constructs. Variables with the strongest canonical loadings were retained to define two latent constructs: InflamMetab, an inflammatory-metabolic burden latent factor (CRP, GGT, FBS, and abdominal circumference ~~ratio~~), and ExecFunc, an executive dysfunction latent factor (EXIT-25, TMT-B, RBANS-Visuospatial, and CLOX). Cognitive indicators were retained on their native scales; however, the EXIT-25 and TMT-B, for which higher values indicate poorer performance, were reverse-scored (multiplied by -1) so that higher values consistently reflected better executive performance across indicators (e.g., RBANS-Visuospatial and CLOX). Two residual covariances were specified a priori on substantive grounds and retained between RBANS-Visuospatial and CLOX, reflecting shared visuoconstructional/graphomotor variance, and between FBS and abdominal circumference, reflecting shared adiposity-glycemic metabolic variance. We adjusted for potential confounding by regressing both latent variables on age, sex, ethnicity, education, and income. We included direct paths from age to TMT-B (reverse-scored) and EXIT-25 (reverse-scored) in the primary SEM to account for indicator-specific age effects (i.e., differential item functioning) that are not fully captured by the latent executive function construct. ^30^ Both measures are particularly sensitive to age-related influences such as processing speed, motor/visual scanning demands, and task-specific administration effects; allowing these direct effects to be controlled helps ensure that the latent executive factor reflects the shared executive construct rather than residual age-related method variance.

The SEM was fit across imputed datasets using robust maximum likelihood with sandwich standard errors, and pooled inference followed Rubin’s rules. The structural model specified ExecFunc ~ InflamMetab. InflamMetab was regressed on age, sex, ethnicity, education, and income, and Executive Function was regressed on InflamMetab and the same covariates. Models were estimated using robust maximum likelihood (MLR). Model fit was evaluated using the comparative fit index (CFI ≥ 0.90), Tucker–Lewis index (TLI ≥ 0.90), root mean square error of approximation (RMSEA ≤ 0.05) with 90% confidence intervals, and standardized root mean square residual (SRMR < 0.08). ^31^ Modification indices were inspected to identify localized areas of misfit, with any added residual covariances required to have clear substantive justification (e.g., shared method variance among similar cognitive measures). Standardized estimates were reported alongside unstandardized estimates. Parameter estimates were obtained in each imputed dataset and pooled across imputations. All analyses were conducted in R using lavaan (v0.6-21) and lavaan.mi.

**Supplementary Tables**

**Table S1.** Missing variables

| Variable | Number of missing | Percent missing |
| --- | --- | --- |
| Age | 1 | 0.1 |
| Sex | 6 | 0.4 |
| Ethnicity | 13 | 1.0 |
| Education | 6 | 0.4 |
| Income | 26 | 1.9 |
| Hemoglobin A1c | 74 | 5.5 |
| Fasting glucose | 67 | 4.9 |
| HDL | 67 | 4.9 |
| Triglycerides | 67 | 4.9 |
| SBP | 81 | 6 |
| BMI | 11 | 0.8 |
| Abdominal circumference | 12 | 0.9 |
| Serum creatinine | 67 | 4.9 |
| CRP | 562 | 41.4 |
| GGT | 78 | 5.7 |
| Albumin | 71 | 5.2 |
| RBANS Immediate Memory | 63 | 4.6 |
| RBANS Visuospatial/ Constructional | 84 | 6.2 |
| RBANS Language | 66 | 4.9 |
| RBANS Attention | 79 | 5.8 |
| RBANS Delayed Recall | 75 | 5.5 |
| EXIT-25 | 102 | 7.5 |
| TMT-A | 92 | 6.8 |
| TMT-B | 178 | 13.1 |
| FAS | 86 | 6.3 |
| CLOX | 76 | 5.6 |

C-Reactive Protein-CRP; Gamma-glutamyl transferase-GGT; Fasting Blood Glucose-FBS; Body Mass Index-BMI; High-density lipoprotein-HDL; Repeatable Battery for the Assessment of Neuropsychological Status-RBANS; Executive Interview 25-EXIT 25; Clock Drawing Test-CLOX; Trail Making Test A-TMT-A; Trail Making Test B-TMT-B

**Table S2.** Structural equation model parameter estimates

|  | Estimate | SE | t-value | p value | Std.lv | Std.all |
| --- | --- | --- | --- | --- | --- | --- |
| Latent Variables: | | | | | | |
| InflamMetab =~ |  |  |  |  |  |  |
| CRP | 0.313 | 0.061 | 5.125 | 0 | 0.351 | 0.352 |
| GGT | 0.461 | 0.05 | 9.162 | 0 | 0.517 | 0.518 |
| FBS | 0.277 | 0.044 | 6.257 | 0 | 0.311 | 0.312 |
| Abdominal Circumference | 0.367 | 0.052 | 7.025 | 0 | 0.412 | 0.412 |
| ExecFunc =~ |  |  |  |  |  |  |
| EXIT-25 | 0.355 | 0.022 | 16.383 | 0 | 0.611 | 0.611 |
| TMT-B | 0.439 | 0.024 | 18.62 | 0 | 0.756 | 0.756 |
| RBANS Visuospatial | 0.303 | 0.021 | 14.708 | 0 | 0.522 | 0.523 |
| CLOX | 0.321 | 0.022 | 14.354 | 0 | 0.553 | 0.554 |
| Regressions: | | | | | | |
| ExecFunc ~ InflamMetab | -0.31 | 0.084 | -3.7 | 0 | -0.202 | -0.202 |
| InflamMetab ~ |  |  |  |  |  |  |
| Age | -0.314 | 0.062 | -5.102 | 0 | -0.28 | -0.28 |
| Sex | 0.656 | 0.153 | 4.29 | 0 | 0.584 | 0.272 |
| Ethnicity | -0.124 | 0.159 | -0.78 | 0.436 | -0.111 | -0.054 |
| Education | -0.142 | 0.075 | -1.905 | 0.057 | -0.127 | -0.127 |
| Income | -0.581 | 0.111 | -5.224 | 0 | -0.517 | -0.257 |
| ExecFunc ~ |  |  |  |  |  |  |
| Age | -0.517 | 0.086 | -5.992 | 0 | -0.3 | -0.3 |
| Sex | 0.046 | 0.11 | 0.416 | 0.677 | 0.027 | 0.012 |
| Ethnicity | -0.226 | 0.124 | -1.822 | 0.069 | -0.131 | -0.064 |
| Education | 0.98 | 0.075 | 13.117 | 0 | 0.569 | 0.569 |
| Income | 0.614 | 0.103 | 5.943 | 0 | 0.357 | 0.177 |
| TMT-B ~ |  |  |  |  |  |  |
| Age | -0.192 | 0.035 | -5.416 | 0 | -0.192 | -0.192 |
| EXIT-25 ~ |  |  |  |  |  |  |
| Age | -0.118 | 0.032 | -3.698 | 0 | -0.118 | -0.118 |
| Covariances: | | | | | | |
| .RBANS Visuospatial ~~ |  |  |  |  |  |  |
| .CLOX | 0.09 | 0.023 | 4.003 | 0 | 0.09 | 0.127 |
| .FBS ~~ |  |  |  |  |  |  |
| .Abdominal Circumference | 0.1 | 0.033 | 3.043 | 0.002 | 0.1 | 0.116 |
| Variances: |  |  |  |  |  |  |
| .CRP | 0.875 | 0.055 | 15.83 | 0 | 0.875 | 0.876 |
| .GGT | 0.731 | 0.069 | 10.539 | 0 | 0.731 | 0.732 |
| .FBS | 0.902 | 0.088 | 10.267 | 0 | 0.902 | 0.903 |
| .Abdominal Circumference | 0.829 | 0.053 | 15.531 | 0 | 0.829 | 0.83 |
| .EXIT-25 | 0.572 | 0.032 | 17.925 | 0 | 0.572 | 0.572 |
| .TMT-B | 0.309 | 0.024 | 13.026 | 0 | 0.309 | 0.31 |
| .RBANS Visuospatial | 0.726 | 0.033 | 21.828 | 0 | 0.726 | 0.727 |
| .CLOX | 0.693 | 0.047 | 14.677 | 0 | 0.693 | 0.694 |
| .InflamMetab | 1 |  |  |  | 0.793 | 0.793 |
| .ExecFunc | 1 |  |  |  | 0.337 | 0.337 |
| R-Square: | Estimate |  |  |  |  |  |
| CRP | 0.124 |  |  |  |  |  |
| GGT | 0.268 |  |  |  |  |  |
| FBS | 0.097 |  |  |  |  |  |
| Abdominal Circumferenceabcir | 0.17 |  |  |  |  |  |
| EXIT-25 | 0.428 |  |  |  |  |  |
| TMT-B | 0.69 |  |  |  |  |  |
| RBANS Visuospatial | 0.273 |  |  |  |  |  |
| CLOX | 0.306 |  |  |  |  |  |
| InflamMetab | 0.207 |  |  |  |  |  |
| ExecFunc | 0.663 |  |  |  |  |  |

SE- Standard error

**Supplementary Figures**
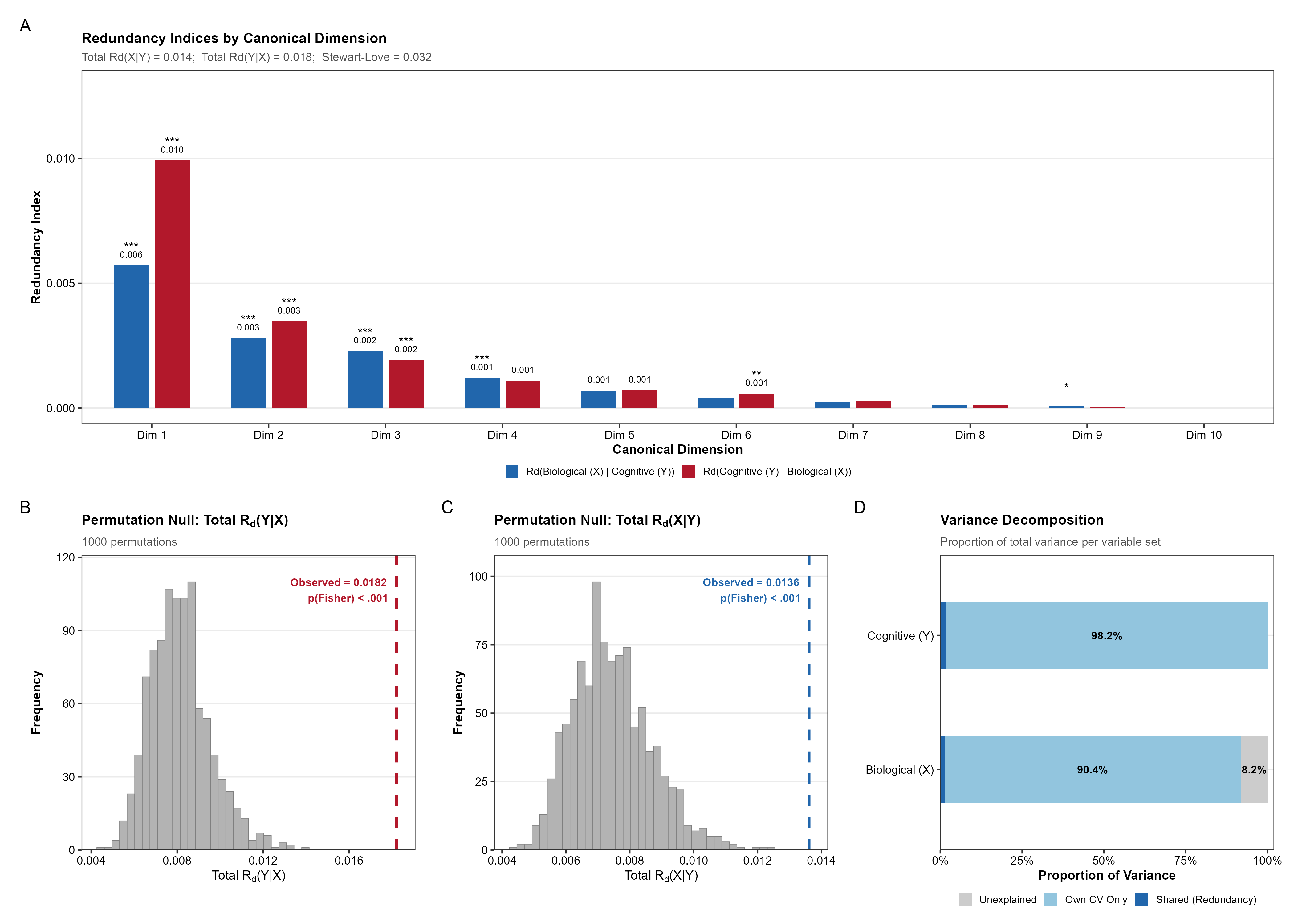


**Figure S1. Redundancy analysis of the canonical correlation between biological and cognitive variable sets.** (A) Redundancy indices by canonical dimension, showing the proportion of variance in each variable set explained by the opposite set's canonical variates. The first three dimensions contributed the most shared variance, with the cognitive set (Y) consistently explaining slightly more variance in the biological set (X) than vice versa. Total redundancy was 1.4% for Rd(X|Y) and 1.8% for Rd(Y|X). (B) Permutation null distribution (1,000 permutations) for the total redundancy index Rd(Y|X). The red vertical line indicates the observed value, which exceeded all permuted values (p < .001), confirming the significance of the multivariate biological–cognitive association. (C) Variance decomposition for each variable set, partitioned into shared variance (redundancy attributable to the opposite set), variance explained exclusively by the set's own canonical variates, and unexplained variance. Redundancy analysis was conducted separately across 20 multiply imputed datasets, with permutation p-values combined using Fisher's method. All variables were residualized on age, sex, and education prior to analysis. Cognitive variables scored in the direction of impairment (Trail Making Tests A and B, EXIT) were sign-reversed so that higher values uniformly indicate better performance.

**References**

(1) Hotelling, H. Relations between two sets of variates. In *Breakthroughs in statistics: methodology and distribution*, Springer, 1992; pp 162-190.

(2) Randolph, C.; Tierney, M. C.; Mohr, E.; Chase, T. N. The Repeatable Battery for the Assessment of Neuropsychological Status (RBANS): preliminary clinical validity. *J Clin Exp Neuropsychol* **1998**, *20* (3), 310-319. DOI: 10.1076/jcen.20.3.310.823 From NLM.

(3) Borkowski, J. G.; Benton, A. L.; Spreen, O. Word fluency and brain damage. *Neuropsychologia* **1967**, *5* (2), 135-140. DOI: 10.1016/0028-3932(67)90015-2.

(4) Royall, D. R.; Mahurin, R. K.; Gray, K. F. Bedside assessment of executive cognitive impairment: the executive interview. *J Am Geriatr Soc* **1992**, *40* (12), 1221-1226. DOI: 10.1111/j.1532-5415.1992.tb03646.x From NLM.

(5) *Army Individual Test Battery. Manual of directions and scoring.*; Washington, DC:  War Department, Adjutant General's Office., 1944.

(6) Ashendorf, L.; Jefferson, A. L.; O'Connor, M. K.; Chaisson, C.; Green, R. C.; Stern, R. A. Trail Making Test errors in normal aging, mild cognitive impairment, and dementia. *Arch Clin Neuropsychol* **2008**, *23* (2), 129-137. DOI: 10.1016/j.acn.2007.11.005 From NLM.

(7) Reitan, R. M. Validity of the Trail Making Test as an indicator of organic brain damage. *Perceptual and motor skills* **1958**, *8* (3), 271-276.

(8) Royall, D. R.; Cordes, J. A.; Polk, M. CLOX: an executive clock drawing task. *J Neurol Neurosurg Psychiatry* **1998**, *64* (5), 588-594. DOI: 10.1136/jnnp.64.5.588 From NLM.

(9) Lin, Y.; Gong, Z.; Ma, C.; Wang, Z.; Wang, K. Relationship between glycemic control and cognitive impairment: A systematic review and meta-analysis. *Front Aging Neurosci* **2023**, *15*, 1126183. From NLM.

(10) Saczynski, J. S.; Jónsdóttir, M. K.; Garcia, M. E.; Jonsson, P. V.; Peila, R.; Eiriksdottir, G.; Olafsdottir, E.; Harris, T. B.; Gudnason, V.; Launer, L. J. Cognitive impairment: an increasingly important complication of type 2 diabetes: the age, gene/environment susceptibility--Reykjavik study. *Am J Epidemiol* **2008**, *168* (10), 1132-1139. DOI: 10.1093/aje/kwn228 From NLM.

(11) Solomon, A.; Kivipelto, M.; Wolozin, B.; Zhou, J.; Whitmer, R. A. Midlife serum cholesterol and increased risk of Alzheimer's and vascular dementia three decades later. *Dement Geriatr Cogn Disord* **2009**, *28* (1), 75-80. DOI: 10.1159/000231980 From NLM.

(12) Solomon, A.; Kåreholt, I.; Ngandu, T.; Wolozin, B.; Macdonald, S. W.; Winblad, B.; Nissinen, A.; Tuomilehto, J.; Soininen, H.; Kivipelto, M. Serum total cholesterol, statins and cognition in non-demented elderly. *Neurobiol Aging* **2009**, *30* (6), 1006-1009. DOI: 10.1016/j.neurobiolaging.2007.09.012 From NLM.

(13) Cui, Y.; Li, C.; Ke, B.; Xiao, Y.; Wang, S.; Jiang, Q.; Zheng, X.; Lin, J.; Huang, J.; Shang, H. Protective role of serum albumin in dementia: a prospective study from United Kingdom biobank. *Front Neurol* **2024**, *15*, 1458184. DOI: 10.3389/fneur.2024.1458184 From NLM.

(14) Elias, M. F.; Elias, P. K.; Sullivan, L. M.; Wolf, P. A.; D'Agostino, R. B. Obesity, diabetes and cognitive deficit: The Framingham Heart Study. *Neurobiol Aging* **2005**, *26 Suppl 1*, 11-16. DOI: 10.1016/j.neurobiolaging.2005.08.019 From NLM.

(15) Li, J.; Jiao, M.; Wen, J.; Fan, D.; Xia, Y.; Cao, Y.; Shi, R.; Xiao, C. Association of body mass index and blood lipid profile with cognitive function in Chinese elderly population based on data from the China Health and Nutrition Survey, 2009-2015. *Psychogeriatrics* **2020**, *20* (5), 663-672. DOI: 10.1111/psyg.12559 From NLM.

(16) Norris, T.; Salzmann, A.; Henry, A.; Garfield, V.; Pinto Pereira, S. M. The relationship between adiposity and cognitive function: a bidirectional Mendelian randomization study in UK Biobank. *Int J Epidemiol* **2023**, *52* (4), 1074-1085. DOI: 10.1093/ije/dyad043 From NLM.

(17) Kałamała, P.; Ware, N.; Fabiani, M.; Michie, P.; Hunter, M.; Wade, A.; Simpson, F.; Mellow, M. L.; Low, K.; Keage, H. A. D.; et al. Cardiorespiratory fitness and cardiometabolic health are associated with distinct cognitive domains in cognitively healthy older adults. *Sci Rep* **2025**, *15* (1), 42849. DOI: 10.1038/s41598-025-26105-x From NLM.

(18) Williamson, J. D.; Pajewski, N. M.; Auchus, A. P.; Bryan, R. N.; Chelune, G.; Cheung, A. K.; Cleveland, M. L.; Coker, L. H.; Crowe, M. G.; Cushman, W. C.; et al. Effect of Intensive vs Standard Blood Pressure Control on Probable Dementia: A Randomized Clinical Trial. *Jama* **2019**, *321* (6), 553-561. DOI: 10.1001/jama.2018.21442 From NLM.

(19) Ravaglia, G.; Forti, P.; Maioli, F.; Chiappelli, M.; Montesi, F.; Tumini, E.; Mariani, E.; Licastro, F.; Patterson, C. Blood inflammatory markers and risk of dementia: The Conselice Study of Brain Aging. *Neurobiol Aging* **2007**, *28* (12), 1810-1820. DOI: 10.1016/j.neurobiolaging.2006.08.012 From NLM.

(20) Long, S.; Chen, Y.; Meng, Y.; Yang, Z.; Wei, M.; Li, T.; Ni, J.; Shi, J.; Tian, J. Peripheral high levels of CRP predict progression from normal cognition to dementia: A systematic review and meta-analysis. *J Clin Neurosci* **2023**, *107*, 54-63. DOI: 10.1016/j.jocn.2022.11.016 From NLM.

(21) van den Kommer, T. N.; Dik, M. G.; Comijs, H. C.; Jonker, C.; Deeg, D. J. Role of lipoproteins and inflammation in cognitive decline: do they interact? *Neurobiol Aging* **2012**, *33* (1), 196.e191-112. DOI: 10.1016/j.neurobiolaging.2010.05.024 From NLM.

(22) Gasparini, F.; Valletta, M.; Vetrano, D. L.; Beridze, G.; Rizzuto, D.; Calderón-Larrañaga, A.; Fredolini, C.; Dale, M.; Winblad, B.; Fratiglioni, L.; et al. Kidney Function, Alzheimer Disease Blood Biomarkers, and Dementia Risk in Community-Dwelling Older Adults. *Neurology* **2026**, *106* (1), e214446. DOI: 10.1212/wnl.0000000000214446 From NLM.

(23) Chen, J.; Liang, C.; Liu, C.; Jie, L.; Liu, B.; Yang, X. Liver enzyme and risk of vascular dementia: A univariable and multivariable Mendelian randomization of European descent. *Neuroprotection* **2024**, *2* (4), 310-317. DOI: 10.1002/nep3.67 From NLM.

(24) Zhong, Y.; Li, L. Association between liver biomarkers and risk of cognitive impairment and dementia: A systematic review and meta-analysis. *Pak J Med Sci* **2025**, *41* (7), 2122-2132. DOI: 10.12669/pjms.41.7.12321 From NLM.

(25) Chen, M. D.; Deng, C. F.; Chen, P. F.; Li, A.; Wu, H. Z.; Ouyang, F.; Hu, X. G.; Liu, J. X.; Wang, S. M.; Tang, D. Non-invasive metabolic biomarkers in initial cognitive impairment in patients with diabetes: A systematic review and meta-analysis. *Diabetes Obes Metab* **2024**, *26* (12), 5519-5536. DOI: 10.1111/dom.15916 From NLM.

(26) Huang, Y. Y.; Wang, H. F.; Wu, B. S.; Ou, Y. N.; Ma, L. Z.; Yang, L.; Cheng, W.; Yu, J. T. Clinical laboratory tests and dementia incidence: A prospective cohort study. *J Affect Disord* **2024**, *351*, 1-7. DOI: 10.1016/j.jad.2024.01.226 From NLM.

(27) Han, L.; Li, Q.; Zhang, L.; Yu, J.; Liu, Y.; Li, W.; Ping, F.; Zhang, H.; Li, Y.; Xu, L. The necessity of strengthening glycemic and lipid metabolism management for improving brain structure and cognitive function in people with diabetes: A retrospective study based on UK Biobank. *Diabetes Res Clin Pract* **2025**, *226*, 112366. DOI: 10.1016/j.diabres.2025.112366 From NLM.

(28) Van Buuren, S.; Groothuis-Oudshoorn, K. mice: Multivariate imputation by chained equations in R. *Journal of statistical software* **2011**, *45*, 1-67.

(29) Karpman, M. B. Redundancy in canonical analysis. *Research Quarterly for Exercise and Sport* **1981**, *52* (2), 291-292.

(30) Cheng, Y.; Shao, C.; Lathrop, Q. N. The Mediated MIMIC Model for Understanding the Underlying Mechanism of DIF. *Educ Psychol Meas* **2016**, *76* (1), 43-63. DOI: 10.1177/0013164415576187 From NLM.

(31) Hu, L. t.; Bentler, P. M. Cutoff criteria for fit indexes in covariance structure analysis: Conventional criteria versus new alternatives. *Structural equation modeling: a multidisciplinary journal* **1999**, *6* (1), 1-55.
